# Prospects for novel inhibitors of peptidoglycan transglycosylases

**DOI:** 10.1016/j.bioorg.2014.05.007

**Published:** 2014-08

**Authors:** Nicola F. Galley, Amy M. O’Reilly, David I. Roper

**Affiliations:** School of Life Sciences, University of Warwick, Coventry CV4 7AL, UK

**Keywords:** Glc*N*Ac, *N*-acetyl-glucosamine, Mur*N*Ac, *N*-acetyl-muramic acid, Gal*N*Ac, *N*-acetyl-galactosamine, UDP, uridine diphosphate, PBP, penicillin binding protein, Peptidoglycan, Transglycosylase, Inhibitor, Antibiotic, Discovery

## Abstract

The lack of novel antimicrobial drugs under development coupled with the increasing occurrence of resistance to existing antibiotics by community and hospital acquired infections is of grave concern. The targeting of biosynthesis of the peptidoglycan component of the bacterial cell wall has proven to be clinically valuable but relatively little therapeutic development has been directed towards the transglycosylase step of this process. Advances towards the isolation of new antimicrobials that target transglycosylase activity will rely on the development of the enzymological tools required to identify and characterise novel inhibitors of these enzymes. Therefore, in this article, we review the assay methods developed for transglycosylases and review recent novel chemical inhibitors discovered in relation to both the lipidic substrates and natural product inhibitors of the transglycosylase step.

## Highlights

• We examine key aspects of transglycosylase inhibitor design.
• Low to high throughput assays suitable for transglycosylase drug discovery.
• Existing chemical start points for transglycosylase active site targeting.

## Graphical abstract

**Figure:**
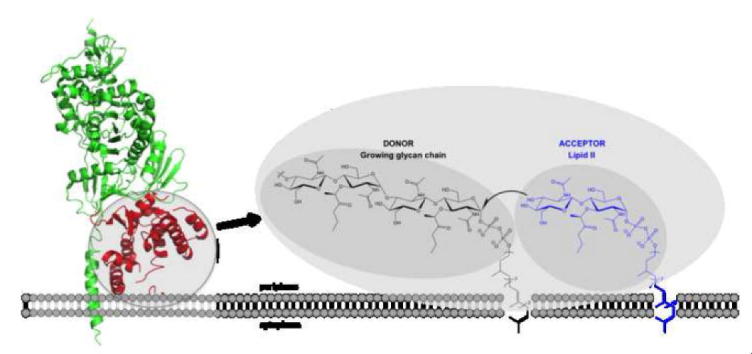

## 1 Introduction

**Fig. 1:**
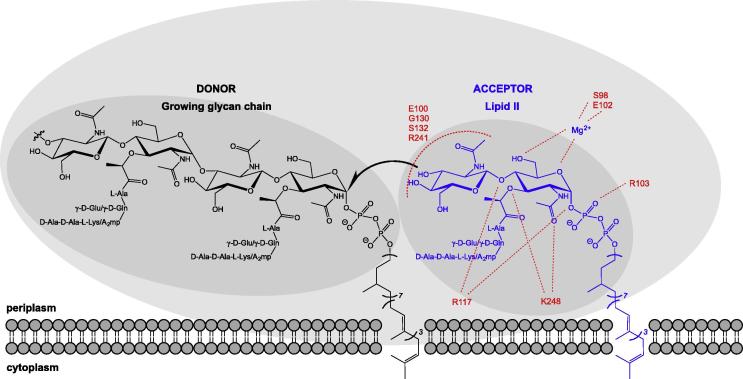
Schematic diagram of the transglycoylase active site showing doner and acceptor sites. Residue numbers in the acceptor sites refer to those determined for *S. aureus* monofunctional transglycosylase in relation to lipid II analogue as described by Huang et al. [28].

In the search for new treatments of bacterial infections and to combat the increasing threat of resistance to existing antimicrobials, there is renewed interest in the exploitation of existing validated targets with novel approaches. With respect to bacterial cell wall biosynthesis, the validity of the peptidoglycan biosynthetic apparatus is well established, particularly in consideration of the fact that many of these antimicrobial targets exist at or beyond the extra-cytoplasmic surface of the cell membrane and are well conserved across all bacterial species [1,2]. The biosynthetic pathway leading to peptidoglycan precursor lipid II and the generalised scheme for its polymerisation into the peptidoglycan layer is well documented. Briefly, uridine 5′-pyrophosphoryl-N-acetyl muramyl-l-alanyl-γ-d-glutamyl-meso-diaminopimelyl-d-alanyl-d-alanine (UDP-Mur*N*Ac-L-Ala-D-Glu-L-(Lys/meso-DAP)-D-Ala-D-Ala) or its l-lysine derivative (UDP-Mur*N*Ac-L-Ala-D-Glu-L-(Lys)-D-Ala-D-Ala) is produced in the cytoplasmic pathway before linkage at the cytoplasmic membrane surface to an undecaprenyl (C55) carrier lipid, prior to the addition of Glc*N*Ac, forming lipid II [3]. This peptidoglycan precursor is then transferred to the outer surface of the cytoplasmic membrane where it is polymerised by monofunctional transglycosylases and class A bifunctional Penicillin Binding Proteins (PBPs) into long glycan chains [4] (Fig. 1). The transpeptidase activity of Class A and B PBPs then produce inter-strand peptide cross-links from pentapeptides emanating from adjacent glycan chains. The resulting polymer has the mechanical strength and rigidity required to resist cytoplasmic osmotic stress and forms a scaffold for a number of extracellular structures and functions.

Both academic and industrial effort over many decades has been directed towards the transpeptidase function of the penicillin binding proteins (PBPs) in this context, with the development of many generations of β-lactam-based antibiotics [5]. However, there has been relatively little development directed towards the essential transglycosylase function required to provide the polymeric transpeptidase substrate, which can also be the product of the same bifunctional peptidoglycan biosynthetic enzyme [6]. Dual inhibition of both transglycosylase and transpeptidase functions would be a powerful antimicrobial strategy providing therapeutic options in a variety of scenarios, including those currently untreatable. Since the active site of the transglycosylase enzymes exist at the membrane surface where peptidoglycan intermediates are presented to the enzymes, this has been viewed as a difficult interface to target [7]. In addition, consideration of the catalytic function of the enzymes leads to the conclusion that the transglycosylase enzymes have long extended active sites, which traditionally have been viewed as more difficult to target [8]. Nevertheless, nature has already provided an exemplar solution to this issue in the form of the moenomycin group of antimicrobials, which appear to mimic the polymerised form of the substrate within the transglycosylase active site. Poor pharmacokinetics prohibits the use of moenomycins in humans, yet this group of antibiotics has been used for decades in agriculture, principally in animal husbandry applications [9]. Remarkably, there is almost no incidence of resistance to these compounds, which implies that the transglycosylase activity may have significant attraction for future targeting.

Understanding the active site architecture of the transglycosylase through X-ray crystallographic analysis along with advances in biochemical study through the provision of native substrate and chemically defined probes, and the development of assay technologies that can support industry standard screening techniques, provide a new prospect for inhibitor discovery for new generation chemotherapy (Fig. 1). In this review article we provide a perspective of the assay technologies available and compounds recently discovered, that are pertinent in that context.

## 2 Assays for transglycosylase activity

**Fig. 2:**
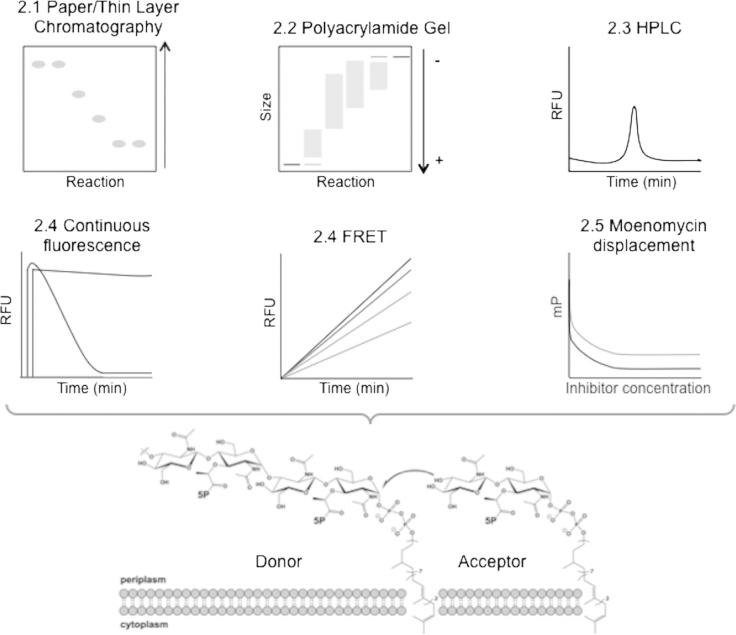
Schematic of the main techniques currently available to assay transglycosylase activity allowing inhibitor discovery as discussed in Section 2. A cartoon representation of a typical reaction trace is shown for each technique and section numbers corresponding to the text are included.

Bacterial transglycosylases have been studied for over 50 years [10]. The discovery and development of novel transglycosylase inhibitors has been highly dependent on appropriate activity assays. However, progress has been hampered by the lack of quantitative and high throughput approaches capable of fast, accurate enzyme activity measurement. In addition, such efforts have been affected by the relative chemical complexity and lack of availability of the transglycosylase substrate, lipid II. Chemical and chemi-enzymatic approaches to overcome this hurdle have been reported by several groups, [11–22]. In addition, lipid II and other peptidoglycan intermediates have become available from the UK Bacterial Cell Wall Biosynthesis Network (UK-BaCWAN). Since both the transglycosylase enzymes and substrate are within a lipid membrane environment, assay conditions and design needs to factor in these chemical properties and physical limitations. The solution of several X-ray crystal structures of mono-functional and bifunctional enzymes has enhanced structure based drug design efforts [7,23–28], an advance which has depended upon the design and implementation of reliable and accurate high-throughput assays. The following sections discuss the main assay types currently available, whilst Fig. 2 and Table 1 provide concise summaries.

### 2.1 Paper and thin layer chromatography

Paper chromatography was first used to study the full polymerisation of peptidoglycan using particulate enzyme preparations isolated from *Staphylococcus aureus* with radiolabelled UDP-*N*-acetylmuramyl-pentapeptide and UDP-*N*-acetylglucosamine as substrates [29]. The assay was adapted to use [^14^C]-labelled lipid II with membrane protein preparations [22]. The use of penicillin to inhibit transpeptidase and carboxypeptidase activities of bifunctional PBPs facilitated analysis of the transglycosylation reaction, and the assay has been used for several studies [30–34]. Whilst scintillation counting allows collection of quantitative data in a stopped assay format, paper chromatography remains a cumbersome technique that is low throughput and lacks the ability to rapidly characterise the product post reaction.

Thin layer chromatography has also been used to study polymerisation, using fluorescent lipid II for detection [35]. With the fluorescent substrate, a dansyl reporting group was linked to the ε-amino group of the lysine side chain of the pentapeptide via a sulfonamide linkage to generate fluorescent dansyl lipid II. Transglycosylase kinetic parameters have been shown to be unaffected by the presence of this group (see Section 2.6 for further discussion).

The presence of a dansyl group prevents transpeptidation from occurring on this molecule, which results in a transglycosylation-specific assay when used as the sole substrate in a stopped assay format. Paper and thin layer chromatography are both highly sensitive techniques, allowing very small amounts of material to be detected. However, the assay remained inherently low throughput and qualitative.

### 2.2 Polyacrylamide gel based techniques

Transglycosylase activity can also be studied using a polyacrylamide gel based assay developed from a technique initially used in the late 1980s. Tricine–SDS–PAGE [36,37] is a variation on the more commonly used glycine SDS-PAGE that has been optimised for low molecular weight proteins. Glycan products made of repeating disaccharide units have a net negative charge, allowing their separation by electrophoresis, and shorter chain lengths in particular are within the optimum separation size range. The system was modified to separate the polymeric products of isolated transglycosylase domains using [^14^C]-lipid II and lipid IV as substrates on a 9% acrylamide gel [38]. In additional experiments, full-length PBPs were used along with penicillin G to inhibit transpeptidase activity. These assays were able to detect the presence of polymeric products, and also allowed visualisation of a range of glycan chain lengths for the first time. Fluorescently labelled lipid II, as discussed above, can also be used as a substrate in gel-based assays [39].

The main strength of this technique is the unique ability to visualise discrete lengths of polymerised material. However this is only possible for shorter chain lengths, with longer polymerised material forming an unresolved smear or high molecular weight product, which does not enter the resolving gel phase [38,39], thus the techniques is not particularly sensitive. The main limitation with this approach is the lack of quantitative data in a continuous assay format. Unpolymerised and polymerised products can be quantified using densitometry for both fluorescent and radioactive material, and rates can be crudely estimated using time-course experiments. Nonetheless, gel-based assays are useful for studying the processivity of enzymes, and they allow simple comparison between different enzymes, which other systems cannot do at present. It has been reported that separation of glycan chains using this approach is not affected by other proteins, salts or additives in reaction buffers [38].

### 2.3 High-pressure liquid chromatography

High-pressure liquid chromatography (HPLC) has been used to separate native muropeptides extracted from bacteria [40] and adapted to identify the products of *in vitro* transglycosylase activity using suitably labelled fluorescent lipid II intermediates created either pre or post reaction [18,41] In the method described by Schwartz et al. 2001, reactions were in the presence of Penicillin G and products are labelled post reaction with fluorescamine via the ε-amino of lysine in the lipid II pentapeptide side chain before being separated by anion exchange [18]. Size exclusion chromatography has been used to separate mixtures of unlabelled and Alexa 647-fluor labelled lipid II substrates and polymerised products [26]. In these cases the reaction products were applied directly to the column with no requirement for sample preparation, although *N*-acetylmuramidase digestion could also be used prior to separation [28]. When the lipid II substrate is radiolabelled, this technique can be adapted to monitor both transglycosylase and transpeptidase products of the reaction by appropriate post reaction enzymatic treatment of the resulting polymer [42,43] since the ε-group of lysine or DAP at position 3 of the pentapeptide stem is free to participate in transpeptidation. HPLC assays in this context are reasonably sensitive, allowing low levels of material to be detected, although sensitivity is dependent on the exact equipment being used and its capabilities.

### 2.4 Fluorometric continuous assays

The first continuous, coupled assay of transglycosylase activity reported was based upon the increased quantum yield of fluorescence signal from a dansyl fluorophore when in a hydrophobic micellar environment [41]. Under the assay conditions used, dansyl lipid II (see Section 2.1) is presented to the transglycosylase in detergent micelles, and can be polymerised into glycan chains. Whilst *N*-acetylmuramidase digestion of the glycan chains generates aqueous soluble labelled monomers, resulting in a reduction in fluorescence as the environment of the fluorophore changes from hydrophobic micellar environment to the soluble phase. The initial rate of this decreased fluorescence was attributed to incorporation of lipid II into glycan chains and was used to determine kinetic parameters for *Escherichia coli* PBP1b transglycosylase activity [41]. The presence of the dansyl group in the third position of the lipid II pentapeptide, prevented subsequent transpeptidation by bifunctional enzymes, allowing measurement of transglycosylation alone. This assay [41] has been converted to a multi-well format, which enables the rapid parallel screening of a range of reaction conditions [44]. This can allow, therefore, the screening and determination of optimal conditions for multiple transglycosylases from a range of microorganisms, essential in the study of these membrane proteins. In addition, this demonstrated the basis for utility of this assay in library screening of compounds to identify potential novel inhibitors, as did a second study [45].

Whilst measuring changes in fluorescence serve well for efficient enzyme and inhibitor characterisation, they are not always suitable for high throughput, pharmaceutical industry standard, compound screening. Thus, the development of time resolved Förster Resonance Energy Transfer (FRET) assays is of interest to address these requirements since it is possible to avoid contaminating fluorescence signals from compounds within the libraries screened. Huang et al. utilised a FRET-Based Lipid II Analogue (FBLA), with a Coumarin fluorophore in the third position of the peptide stem and a dimethylamino-azobenzenesulfonyl quencher in the lipid chain of the same substrate molecule [46]. Prior to polymerisation of the FBLA, the coumarin fluorescence is quenched, but once polymerised into glycan chains, the quencher is lost as the polyprenyl lipid tail is released from the transglycosylase-substrate complex, and the polymerised glycan product is fluorescent. Inclusion of *N*-acetylmuramidase to digest the glycan product into smaller and more soluble intermediates enhanced the fluorescence changes observed. When adapted to a 1536-well format, a very high throughput approach was achieved, and a library of 120,000 compounds was screened. A number of previously characterised inhibitors including moenomycin were identified in screens utilising a variety of Class A PBPs (including *Acinetobacter baumannii* PBP1b, *Clostridium difficile* PBP, *E. coli* PBP1b, *Klebsiella pneumoniae* PBP1b, and *Mycobacterium tuberculosis* PonA1). This was the first application of FRET in the study of transglycosylation, and represents a new sensitive method for continuously following transglycosylation activity.

The high throughput nature of these assays is a clear advantage, and the ability to continuously monitor enzyme activity and accurately determine kinetic parameters for a wide range of conditions and enzymes makes this a powerful approach. Both types of fluorometric assay described here measure overall transglycosylation rates, and are capable of identifying inhibitors which interfere with substrate availability as well as those that directly inhibit enzyme activity, which may increase the range of possible lead compounds identified. However, both assays use a modified lipid II substrate that differs from the native substrate by addition of large fluorescent groups, and this will be discussed further in Section 2.6.

### 2.5 High throughput screening based on moenomycin displacement

A different approach to high throughput assay design has been taken by a number of groups, based upon chemically modified derivatives of moenomycin, which binds to the transglycosylase active site with high affinity.

Following a surface plasmon resonance (SPR) study of various immobilised Class A PBPs binding to moenomycin, Cheng et al. designed a highly sensitive fluorescence anisotropy based assay utilising a fluorescein labelled moenomycin (F-Moe) [47]. When bound to the transglycosylase active site F-Moe displayed fluorescence anisotropy properties which decreased when the F-Moe was displaced by compounds binding competitively to the active site at comparable or greater affinity. Of all class A PBP homologs tested, it was found that a combination of *Helicobacter pylori* PBP1a with F-Moe had a Kd of 25 (±14) nM and anisotropy reaching 0.2 upon binding of the ligand. This high throughput assay was used to screen 57,000 compounds with a *Z*′ value of 0.895, which proved valuable as a robust initial screen and identified a number of moenomycin derivatives and small molecules, with 3 small molecule hits showing both antibacterial and transglycosylase inhibitory action (HTS6-8) (Table 2). The major limitation of this approach is the inability of compounds with relatively low affinity to displace high affinity moenomycin, precluding for example fragment based drug design or delineation of structure activity relationships of transglycosylase inhibitors.

Gampe et al. described a fluorescence polarisation, displacement assay based upon the binding of a fluorescently labelled, truncated analogue of moenomycin that displayed weaker binding than moenomycin A [48]. This fluorescent probe represented a minimal pharmacophore to specifically identify low micromolar inhibitor binding to the transglycosylase active site and was designed from a consideration of the X-ray crystal structures of moenomycin in complex with transglycosylase active sites. This probe was used in 1536-well plate format using non-essential *S. aureus* monofunctional transglycosylase enzyme SgtB [49], against 110,000 compounds in the Harvard Medical School screen, resulting in a *Z*′ value of 0.78 and initially identifying 186 hits [48]. After dose response studies and the elimination of fluorescent compounds from the initial hits, a number of leads were identified including one (Compound 10) with inhibition constants ranging from 2.6 mM to 95 nM against transglycosylase enzymes from *S. aureus*, *Enterococcus faecalis*, and *E. coli* (Table 2).

Both the assays described by Cheng et al. [47] and Gampe et al. [48] utilise the properties of existing drug-enzyme interaction for the basis of detection, eliminating the need for lipid II substrates in the primary screen and the detection of the reaction products. Both are also sensitive methods, which can detect low levels of displacement. However these assays rely on chemical modification of the pharmacophore for detection in a fluorescence mode and the ability of library compounds to displace existing interactions between enzyme and that pharmacophore.

### 2.6 Prospects for assay development in transglycosylase inhibitor discovery

The transglycosylase activity of purified, recombinant PBPs is highly sensitive to the conditions *in vitro*. In particular: temperature, DMSO, detergents and divalent cations can have significant effects [41,44,50,51]. Also, despite recent advances in membrane protein biochemistry, the level of understanding of the separate functions of Class A PBPs and MGTs and more importantly, their coordinated activity with other cell wall biosynthetic proteins as well as cell division is still somewhat lacking. Additionally, studies have shown that the transmembrane portion of the PBPs may be highly involved in substrate binding as well as in transglycosylase activity [27,28,39]. In fact, enzyme activities of full-length enzymes are often higher than the truncated forms, which supports this hypothesis. Assaying full-length enzymes in a membrane environment and in the presence of a full complement of cell wall proteins may be a highly significant factor in future inhibitor discovery, and this is undoubtedly highly challenging.

It has been demonstrated that transglycosylase kinetic parameters are largely unaffected by addition of side groups on the third position lysine or DAP of the lipid II pentapeptide [16,41]. The effects of fluorophores on the lipid and peptide chains were compared, and those on the lipid chain appear less likely to interfere with enzyme-substrate recognition [52]. Even taking this into consideration, the kinetic parameters measured still appear too low to meet the demands of growing and dividing cells. In order to support growth, an *E. coli* cell would require 300 transglycosylase reactions/minute/molecule, but published data on *E. coli* PBP1a (as a representative example) *in vitro* gave only 0.8 transglycosylase reactions/minute/molecule [53]. Thus, there is a significant gap between observed *in vitro* behaviour and that required to support life *in vivo*, leading to the hypothesis that other regulatory and coordinating factors are necessary for a more accurate reflection of transglycosylase activity during *in vitro* analysis [54].

Taking all of the above into account, it may be desirable to measure alternative product release, rather than polymerised product, to follow transglycosylase reactions [13]. Despite this approach being unsuccessful to date, the concept could pave the way for new ideas on how to assay these biologically and pathophysiologically important enzymes. Eventually it may be advantageous to move away from utilisation of highly modified substrates for enzyme characterisation, and minimise the differences from the physiological substrates. Furthermore, it is apparent that PBPs function in a coordinated manner, and it may be intuitive to include multiple enzymes in assays. Several groups have developed high throughput assays of the coupled transglycosylase-transpeptidase activities of peptidoglycan synthesis using membranes as a source of PBPs [50,55,56], in which the transglycosylases can make many of the interactions they would make *in vivo*, and are thus being studied in a more physiologically relevant environment.

## 3 Known inhibitors of peptidoglycan transglycosylase enzymes

As previous eluded to, peptidoglycan transglycosylases are under-exploited as antimicrobial drug targets, despite their key and clear role in an area of bacterial metabolism that is a validated target for a number of existing antibiotics. Moreover, since most pathogens have at least two transglycosylase enzymes required for peptidoglycan biosynthesis which utilise the same mechanism, resistance to novel compounds would require simultaneous and multiple compensatory mutations. Additionally, the fact that the natural product moenomycin has been used for several decades in agriculture without reports for resistance [57], suggests promise in the search for novel inhibitors targeting the same mechanism, but requiring more acceptable pharmacokinetic properties for human use. The structure, biosynthesis and chemical properties of moenomycin have been reviewed extensively in the recent past [9] and will not form part of this discussion except for reference to the binding site within the transglycosylase active site. The transglycosylases are processive enzymes utilising a donor site in which the growing glycan chain resides anchored to the membrane by the undecaprenyl chain of the previously appended lipid II, and an adjacent acceptor site for the incoming lipid II monomer. As a result, the enzyme active site is comparatively long and extended and must accommodate at least four sugar binding sites. Similar active site architecture is seen in lysozyme which also binds alternating *N*-acetylglucosamine and *N*-acetylmuramic acid repeat units [8]. In the following section, we summarise the latest advancements in inhibitors of transglycosylation, including those based on moenomycin and its analogues, as well as analogues of lipid II.

### 3.1 Moenomycin: The ‘blueprint’ transglycosylase inhibitor

**Fig. 3:**
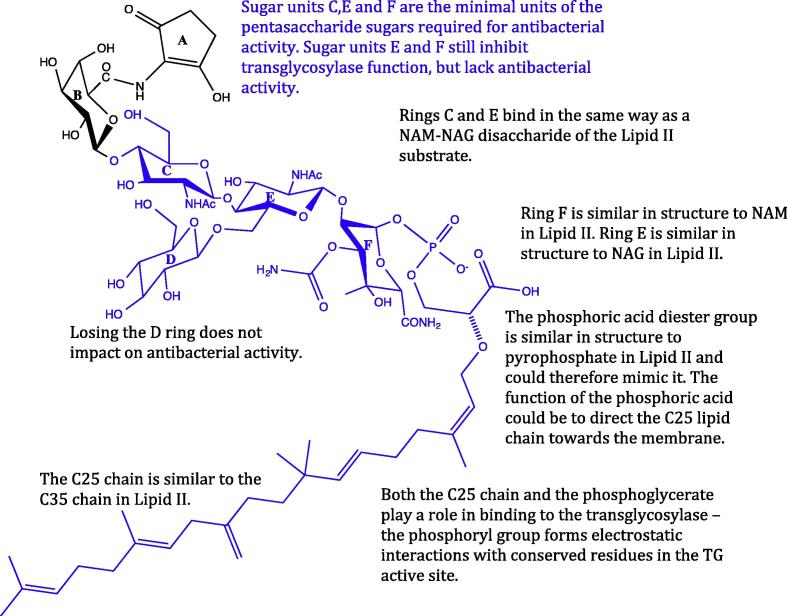
The structure of Moenomycin A, the only known potent inhibitor for bacterial transglycosylases. The region highlighted in blue is the minimal inhibitory pharmacophore, which is often used as a scaffold for the design of new potential inhibitors (discussed in Section 3.1).

The moenomycins are a family of glycolipid antibiotics naturally produced as a complex of related compounds by *Streptomyces ghananensis* with moenomycin A representing the major component with antimicrobial activity [9,58]. Moenomycin consists of a pentasaccharide of units B, C, D, E and F with a chromophore (unit A) and a C25 lipid chain connected to the F saccharide via a phosphoglycerate linker (see Fig. 3). The C25 chain is required for antimicrobial action and in essence the moenomycin structure resembles that of the lipid IV product formed within the transglycosylase active site [59].

Moenomycin has amphiphilic properties due to the hydrophilic nature of the A to F carbohydrate units, the phosphate group of the phosphorglycerate linker and the folded hydrophobic domain formed by the lipid chain. Structure-activity relationships have been carried out on the moenomycin A molecule through selective degradation of its structure and the synthesis of di- and trisaccharide analogues, resulting in an understanding of the minimal pharmacophore [9,60,61]. The degradation of moenomycin to chemical entities that retain the carbohydrate units C, E and F, can be performed with retention of transglycosylase inhibition and antibacterial activity [62,63]. Degradation to retain only the E and F carbohydrate units, still yields transglycosylase inhibition but with the loss of antibacterial activity [64]. The C25 lipid chain is required to achieve full anti-bacterial activity of moenomycin, but is also the origin of its long half-life and contributes to its poor bioavailability and incompatibility for human consumption [9]. Decreasing the length of the lipid chain slowly reduces the inhibitory ability of moenomycin [63], most likely due to loss of ability to anchor itself into the cytoplasmic membrane. Modifying or truncating the lipid chain improves pharmacokinetic properties but the loss of activity needs to be compensated by maintaining essential polar active-site contacts or by utilisation of other hydrophobic chemophores with acceptable properties.

### 3.2 The binding of moenomycin and lipid II to transglycosylase

The two natural molecules known to bind to the transglycosylase domain of PBPs are moenomycin and lipid II. The structural differences between the two must be responsible for inhibition (Fig. 1), as lipid II is the natural substrate for transglycosylation and moenomycin is the most potent inhibitor. Understanding how these two molecules interact and bind to the transglycosylase domain is fundamental in pursuing structural analogues for inhibition.

Moenomycin A binds with high affinity to the transglycosylase domain of several Class A PBPs and is the most potent inhibitor of the transglycosylase function of PBPs with MICs in the region of 0.01–0.1 μg/mL [58]. The mode of inhibition of moenomycin is such that it directly (and reversibly) binds to the active site of the transglycosylase domain, preventing lipid II polymerisation. It first binds to the cytoplasmic membrane *via* its lipid chain, followed by selective binding of the sugar moiety to the donor site of the transglycosylase.

The transglycosylase domain contains five motifs representative of the GT_51_ fold-family [65] and which are conserved among both mono- and bi-functional PBPs. Six residues in the transglycosylase domain have been identified as important in the interaction with moenomycin in *Aquifex aeolicus* PBP1A [24,25], and are conserved across other species. These 6 interactions bind to the F-ring and the phosphoglycerate portion of the drug. The transglycosylase active site is buried in the membrane in order to access the lipid II substrate, explaining the need for a lipid chain on moenomycin A for its inhibitory potency. It is thought that the C25 chain of moenomycin interacts with the transmembrane (TM) segment of *E. coli* PBP1B [47], increasing the binding affinity 5-fold of moenomycin to the transglycosylase domain, highlighting the importance of the TM domain for activity. The regions of moenomycin that make essential contacts with the transglycosylase domain include the C2 on the E ring, C3 on the F ring, the phosphoglycerate moiety and possibly the C10 region of the moenocinol lipid tail. The phosphoryl group and the carboxylate moiety form interactions with conserved active site residues of the transglycosylase domain [66]. The A unit chromophore is not essential for the interaction but may provide higher binding affinity [9].

At the time of writing, the only structural information available for the interaction of lipid II with the transglycosylase domain is that derived from the X-ray crystal structure of *S. aureus* non-functional glycosyltransferase with an analogue of Lipid II [28]. The lipid II analogue used in this study has an undecaprenyl lipid tail, biotinylated ethylene glycol diethyl amine in place of the pentapeptide stem and Gal*N*Ac in place of Glc*N*Ac in the disaccharide moiety and has a Kd of 12.9 μM. Despite the clear importance of the undecaprenyl lipid tail of lipid II for location of the substrate in relation to the enzyme active site, only a discrete section of the disaccharide-pyrophosphate moiety of the lipid II is represented in the electron density at 2.3 Å resolution. The presence of Gal*N*Ac within the lipid II analogue precludes its elongation in the normal transglycosylation reaction, since the position 4 hydroxyl on the sugar ring is in the opposite orientation compared to Glc*N*Ac. but is proposed to bind more tightly to the active site as the distance between that hydroxyl and main-chain carbonyl of G130 is reduced [28].

In consideration of lipid II binding to both the donor and acceptor sites of transglycosylase, lipid II is thought to bind at a lower affinity for the donor site compared to that of lipid IV [14] and this is consistent with the observation of a lag phase in catalysis, seen with many transglycosylase enzymes in the presence of lipid II substrate only. Many inhibitors that bind to transglycosylases occupy the donor site, mimicking the elongating chain of polymerised lipid II [7]. Lovering and co-workers have proposed that moenomycin structurally mimics lipid IV [7,23], a supposition further supported in that lipid IV and moenomycin are suggested to bind to the same site on the transglycosylase domain [67] and that lipid IV may bind to *E. coli* PBP1B with a higher affinity than lipid II [14].

### 3.3 Moenomycin analogues

Finding that the moenomycin degradation products exhibit inhibitory activity against transglycosylases prompted a study in 1999 by Sofia et al., to develop a combinatorial library of 1300 analogues of the moenomycin disaccharide core [68]. These analogues explored modifications at C2 of the E ring and C3 of the F ring (the critical interaction points with the transglycosylase domain). Modifications included aromatic groups attached to the E and F rings and a lipid tail of 12 rather than 25 carbons. Three compounds showed particular promise (Table 2: TS30663, TS30153 and TS30888, with some being more active than the EF disaccharide. These compounds were orders of magnitude less potent than the parent molecule moenomycin, but had IC_50_ values in the range of 10–15 μM [69]. These putative inhibitors also exhibited activity against Gram-positive strains including *Enterococcus faecium* which have tolerance to moenomycin, highlighting that simple degradation compounds show potency against clinically relevant pathogens. The IC_50_ values obtained are within a similar range to other cell wall inhibitors such as bacitracin, vancomycin and ramoplanin. These compounds showed differing activities against transglycosylases in different species, suggesting that they may target different subsets of the transglycosylases [70] as is also seen for β-lactams [71]. A new member of the moenomycin group AC326-α was introduced by He et al. (2000) which has a cyclic moenocinol chain, giving a diumycinol chain [72]. Branched chain lipids show more potent antibacterial activity than linear chained lipids. Putative inhibitors could be designed to mimic moenomycin and could exhibit antibacterial activity to cyclic lipid chains.

Halliday et al. [6] presented a class of compounds from Alchemia, based on the disaccharide scaffold of Sofia et al. [68] with a focus on maintaining the important transglycosylase binding regions. The hits had MIC values of 1–4 μg/mL, against a broad range of Gram-positive organisms [6,68]. One example compound from this class: ACL 19273, showed direct binding and inhibition of the transglycosylase domain, potentially binding to either the acceptor or donor site of the enzyme [6]. Inhibitors that bind to the transglycosylase acceptor site, which may be the case for these small disaccharides, are binding to the contrary site to where moenomycin binds. Determining whether inhibitors bind to the donor or acceptor site of the transglycosylase is important is elucidating their mode of action.

### 3.4 Lipid II analogues

In the early 1990s, efforts were focused on synthesizing transglycosylase inhibitors based on monosaccharide and disaccharide analogues of lipid II, but most were not very active [73–75]. A combination of mono- and disaccharide analogues of lipid II and moenomycin were synthesised by Garneau et al. based on the active part of moenomycin and combining with structural features of lipid II [76]. A lipid II monosaccharide analogue, Compound 5, was designed to mimic the pyrophosphate of lipid II with a dicarboxylate group. Modest activity with just monosaccharide analogues was exhibited, with 28% inhibition of transglycosylases at 100 μM [76]. Clearly the potency of such compounds is weak and it may be that monosaccharide analogues do not have comparable complexity to moenomycin, to sufficiently inhibit transglycosylases [77].

Lipid I and lipid II substrate analogues (both mono- and disaccharides) were synthesised by Terrak and co-workers [77–79] to test the consequences of variations in the lipid chain length, the pyrophosphate and the length of the peptide stem. The disaccharide analogues were 2-fold greater inhibitors than their cognate monosaccharides and as the length of the peptide stem increased from no peptide to l-Ala-d-Glu, to l-Ala-d-Glu- l-Lys, inhibition decreased. This was attributed to the presence of a peptide preventing high affinity binding between the Glc*N*Ac and the transglycosylase. Analogues were tested to highlight important moieties for the future design of substrate-based inhibitors with two hits: C16-phosphoglycerate-Mur*N*Ac-Glc*N*Ac (Compound 21) and C16-phosphoglycerate-Mur*N*Ac-(l-Ala-d-Glu)-Glc*N*Ac (Compound 62). The latter was most active, exhibiting inhibitory activity against the transglycosylase as well as antibacterial ability.

The continuous and quantitative FRET-based assay by Huang and co-workers (as described in Section 2.4) was used to screen a 120,000 compound library containing a variety of bioactive and synthetic molecules, including lipid II analogues [46]. Initially, 25 primary hits were revealed which were subjected to secondary screening. Dose-dependent studies using HPLC and other FRET-based assays identified 7 compounds as transglycosylase inhibitors. The antibacterial activities and MICs were acquired, which showed activity against *S. aureus* and *M. smegmatis*, but not Gram-negative bacteria tested (*E. coli*, *Pseudomonas aeruginosa* and *A. baumannii*). Compounds 19 and 20 were competitive inhibitors of transglycosylase and compounds 24 and 25 were active small molecule inhibitors, with 24 being a previously identified hit [80] with a salicylanilide core structure.

Huang et al. solved the crystal structure of *S. aureus* MGT (SaMGT) in complex with a lipid II substrate analogue, Analogue 3, designed with an inverted 4-OH group on the Glc*N*Ac, which can bind more tightly to the lipid II binding pocket [28]. The compound was used as a donor substrate only, and bound to the SaMGT acceptor site. The purpose of this analogue was primarily as a means to understand binding interactions with the transglycosylase domain, rather than as an inhibitor *per se*.

The role of the pentapeptide moiety in lipid II and its mode of interaction with the transglycosylase has been explored [81]. Lipid II analogues with an assortment of peptide stems were synthesised to analyse their capabilities as transglycosylase substrates. Modifications include incorporating a fluorescent NBD label into position 3 of the peptide or a d-lactyl group at the hydroxyl group of Mur*N*Ac. Surface Plasmon Resonance binding studies were conducted to determine the binding affinity of these analogues to transglycosylase. The three main conclusions from this work were (a) the terminal d-Ala-d-Ala is *not* essential for substrate binding and does not significantly interact with the transglycosylase domain, (b) the fluorescent probe NBD on the ε-amino group of the 3rd position lysine does not affect binding affinity to transglycosylase and (c) the minimum structural requirement for the peptide moiety in lipid II as a transglycosylase is d-lactyl-l-Ala. Further research has shown that only the d-lactyl of the Mur*N*Ac is required for the substrate binding to transglycosylase [78].

### 3.5 Vancomycin derivatives bind to transglycosylase

**Fig. 4:**
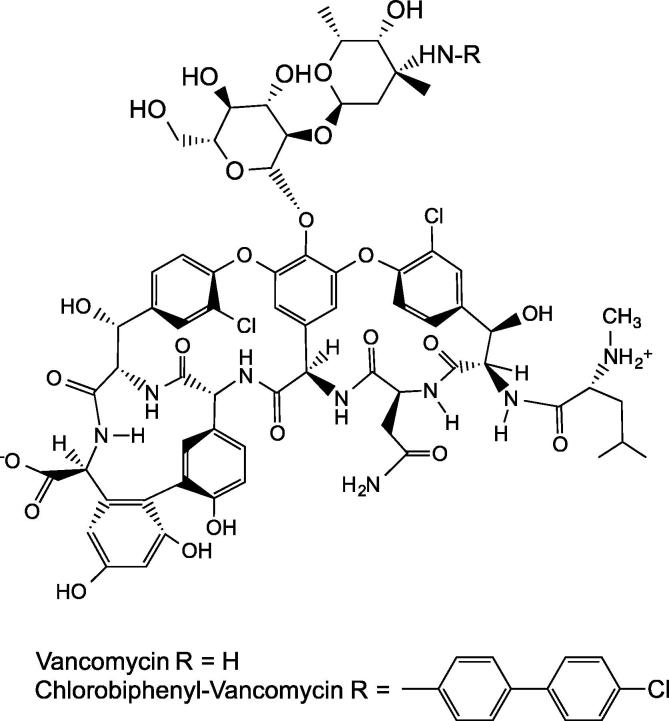
The structure of vancomycin and its derivative chlorobiphenyl vancomycin (CBP-V), which showed antibacterial activity against vancomycin-resistant *Enterococci* (VRE) [85] (discussed in Section 3.5).

The natural product antibiotic vancomycin normally inhibits peptidoglycan polymerisation by binding to the terminal d-Ala-d-Ala moiety on the lipid II pentapeptide stem, inhibiting transpeptidation. Controversially, hydrophobic vancomycin derivatives have been shown to inhibit peptidoglycan polymerisation through preventing transglycosylation, most likely through binding the transglycosylase domain of PBPs and in the absence of dipeptide and depsi-peptide binding [34,82–84]. Common examples of vancomycin derivatives have lipid moieties at the aglycone or on the carbohydrates. One is produced from alkylating vancomycin on the vancosamine sugar with chlorobiphenyl, giving chlorobiphenyl-vancomycin (CBP-V) [85] (Fig. 4). CBP-V showed antibacterial activity against vancomycin-resistant strains, e.g. vancomycin-resistant *Enterococci* (VRE), where the di-peptide moiety in lipid II is substituted for d-Ala-d-lactate.

When the vancomycin N-terminal methyl leucine required for binding d-Ala-d-Ala, was removed from chlorobiphenyl vancomycin (yielding chlorobiphenyl desleucyl-vancomycin), the derivative retained antibacterial activity for both sensitive and resistant bacteria, despite no longer being able to bind its di-peptide ligand [85]. In contrast, when the N-terminal methyl-leucine was removed from full-length vancomycin, it could no longer bind to the d-Ala- d-Ala of lipid II and so was no longer active. The mechanism of action of chlorobiphenyl desleucyl-vancomycin on vancomycin sensitive strains is through either binding the d-Ala-d-Ala of lipid II, or by preventing transglycosylation. Chlorobiphenyl desleucyl vancomycin is missing an important portion of the di-peptide binding pocket [85]. Activity of the vancomycin derivatives decreases when the peptide-binding pockets are damaged [34], suggesting that inhibition is through a mechanism not involving di-peptide binding [84].

## 4 Prospects for new transglycosylase inhibitors

Developing new drugs with antibacterial properties through inhibition of peptidoglycan transglycosylation is of current interest to both academia and the pharmaceutical industry. Currently, most compounds discovered, summarised in Table 2 have greater potency against Gram-positive bacteria than Gram-negative presumably due to accessibility, as is the case with many other targeted compounds. Progress on the development of transglycosylase inhibitors has been slow historically due to complexity of the active site of the enzymes, lack of suitable assays for high throughput screening, provision of suitable substrates for such assays and the difficulties surrounding the reconstitution of activity of these membrane proteins. The availability of lipid II substrate from chemi-enzymatic and total chemical synthesis domains allows transglycosylases from various species to be studied along with a growing literature detailing molecular architecture interactions within the active site. Further understanding of substrate specificity will aid the design of future substrate analogues, common features of which are becoming apparent.

The development of glycolipids and glycopeptides as putative transglycosylase inhibitors has shown that there are new prospects for the combinatorial biosynthesis of phosphoglycolipid antibiotics [86] and there are new generation glycopeptides currently in clinical development that inhibit the transglycosylation process [87]. In addition, research is on-going to determine the exact inhibitory mechanism of moenomycin on transglycosylases, with a drive towards finding novel inhibitory compounds with distinct structural features. Total synthesis of moenomycin A has been achieved [88] and the biosynthetic pathway variants can be theoretically generated which could help in the quest to design new compounds with better pharmacokinetics [89].

We now have the ability to synthesise structurally diverse substrates and to combine synthetic and biological compounds by either enzymatic modification of synthetic analogues or by chemical modification of biosynthetic intermediates. These capabilities enable better comprehension of the role of lipid II in binding to the transglycosylase domain and help to optimise structures for the transglycosylase donor and acceptor sites. These sites have different requirements for lipid chain length, which is important for the processivity of the transglycosylase, with the donor site requiring a C20 lipid chain and the acceptor site tolerating shorter lipids, so there is a compromise between lipid chain length and antibiotic activity [90]. Walker and co-workers have predicted that lipid II with four successive *cis* isoprene units in a 35-carbon chain is the best transglycosylase substrate [21]. Investigating the optimal substrate for transglycosylases such as lipid IV or longer as potential substrate inhibitors may be a worthwhile focus and could be fruitful in generating moenomycin mimics, without the poor pharmacokinetics [14]. Despite the evolution of structurally diverse substrates, there is still more room to understand transglycosylase-substrate mimics.

The structures of transglycosylase domains resemble more closely the structures of glycosidases such as lysozyme, rather than other glycosyl-transferases. Therefore, glycosidases may be better representatives for inhibitor design and lessons may be learned from several decades of experience with the great glycosyl-transferase families [65]. Although there are now in the region of ten structures of transglycosylase enzymes in the protein databank, only a small subset are below 2.5 Å resolution and thus reliable for structure based drug design efforts. This does include however the *S. aureus* monofunctional enzyme in apo (2.5 Å) and lipid II analogue (2.3 Å) forms [28] and the 2.2 Å *E. coli* PBPIb structure in complex with moenomycin [27]. Curiously, the latter structure is monomeric whereas the enzyme in solution has been shown to dimerise at a Kd well below that achieved in both crystallisation and presumable *in-crystallo* [42]. The recent crystal structures of transglycosylase domains in complex with moenomycin have highlighted essential interactions but their significance in structure based drug design efforts must be viewed in the context of the overall processive transglycosylase mechanism.

In the recent past, a number of robust high throughput assays for screening have emerged that provide new prospects for inhibitor discovery. As with all such efforts, the quality and suitability of such libraries must be carefully considered to maximise the potential outputs. The development of a fluorescence polarisation based assay utilising a weaker binding derivative of moenomycin in a displacement assay scenario is particularly encouraging in this respect [48]. Cell based screening assays are also worthy of discussion in this context since they preselect those compounds with the required properties to gain entry to the target cell and are selected on bacteriostatic or bactericidal effects. A set of compounds with a non-carbohydrate, salicylanilide core were identified by Cheng et al. and showed modest inhibitory action against transglycosylases, providing an alternative starting point for medicinal chemical approaches [80].

Further knowledge of the catalytic mechanism and *in vivo* regulation of transglycosylation activity may provide further insight into the chemistry of potential novel lead compounds required for effective chemotherapeutic intervention. Given the renaissance of interest in antimicrobials, the growing concern by public and policy makers regarding antibiotic resistance, the development of new approaches and collaborative efforts between academia and pharma, progress in inhibitor design against transglycosylase may be on the horizon.

## Figures and Tables

**Table 1:** Summary of transglycosylase activity assays as discussed in the text.

Assay type	Section number	Stopped or continuous	Sensitivity	Inhibitor screens
Paper/thin layer chromatography	2.1	Stopped	High	No
Polyacrylamide gel	2.2	Stopped	Low	No
HPLC	2.3	Stopped	Medium	No
Fluorometric: continuous fluorescence	2.4	Continuous	High	Yes
Fluorometric: FRET	2.4	Continuous	High	Yes
Moenomycin displacement	2.5	Continuous	High	Yes

**Table 2:** Reference and chemical structure of transglycosylase inhibitors discussed.

Reference	Compound name/features			Year
Sofia et al. [68]				1999
	TS30153	TS30663		
				
	TS30888			
He et al. [72]				2000
	AC326-α			
Halliday et al. [6]				2006
	ACL 19273			
Cheng et al. [47]				2008
	HTS-6	HTS-7	HTS-8	
Gampe et al. [48]				2013
	Compound 10			
Garneau et al. [76]				2004
	Compound 5			
Cheng et al. [80]				2010
	Compound 24			
Huang et al. [28]				2012
	Analogue 3			
Huang et al. [28]				2012
	Compound 31			
Dumbre et al. [78]				2012
	Compound 21	Compound 62		
Huang et al. [46]				2013
	Compound 19	Compound 20		
				
	Compound 24	Compound 25		
